# Learning to Model Task-Oriented Attention

**DOI:** 10.1155/2016/2381451

**Published:** 2016-05-09

**Authors:** Xiaochun Zou, Xinbo Zhao, Jian Wang, Yongjia Yang

**Affiliations:** ^1^School of Electronics and Information, Northwestern Polytechnical University, Chang'an Campus, P.O. Box 886, Xi'an, Shaanxi 710129, China; ^2^School of Computer Science, Northwestern Polytechnical University, Chang'an Campus, P.O. Box 886, Xi'an, Shaanxi 710129, China

## Abstract

For many applications in graphics, design, and human computer interaction, it is essential to understand where humans look in a scene with a particular task. Models of saliency can be used to predict fixation locations, but a large body of previous saliency models focused on free-viewing task. They are based on bottom-up computation that does not consider task-oriented image semantics and often does not match actual eye movements. To address this problem, we collected eye tracking data of 11 subjects when they performed some particular search task in 1307 images and annotation data of 2,511 segmented objects with fine contours and 8 semantic attributes. Using this database as training and testing examples, we learn a model of saliency based on bottom-up image features and target position feature. Experimental results demonstrate the importance of the target information in the prediction of task-oriented visual attention.

## 1. Introduction

For many applications in graphics, design, and human computer interaction, it is essential to understand where humans look in a scene with a particular task. For example, an understanding of task-oriented visual attention is useful for automatic object recognition [1], image understanding, or image search [2, 3]. It can be used to direct visual search and foveated image video compression [4, 5] and robot localization [6, 7]. It can also be used in advertising design or implementation of smart cameras [8].

However, it is not easy to simulate task-oriented human visual behavior perfectly by machine. Attention is an abstractive concept, and it needs objective metrics for evaluation. Judging the results of experiments by intuitive observation is not precise because different people might focus on different regions of the same scene, even with task. To solve this issue, eye tracker equipment pieces that can record human eye fixation, saccades, and gazes are routinely used. Investigations of human eye movement data provide more objective ground truth for studies on computational attention models. At the present time, there are over two dozen databases with eye tracking data for both images and videos in the public domain [9], which mainly focus on “free-viewing” eye movements.

Most existing computational visual attention saliency models have often been evaluated against predicting human fixations in free-viewing task, in which some are biologically inspired and based on a bottom-up computational model and others combine both bottom-up image based saliency cues and top-down image semantic dependent cues. Though the models do well qualitatively, the models have limited use because they frequently perform well only in the context-free scenario.

Motivated by this, we make two contributions in this paper. The first is a large database of task-oriented eye tracking experiments with labels and analysis and the second is a supervised learning model of saliency which combines both bottom-up image based saliency cues and task-oriented image semantic dependent cues. Our database consists of eye tracking data from 11 different users across 1307 images. To our knowledge, it is the first time that such an extensive collection of task-oriented eye tracking data is available for quantitative analysis. For a given image, the eye tracking data is used to create a “ground truth” saliency map which represents where viewers actually look with a particular search task. We introduce a set of bottom-up image features and target position features to define salient locations and use a linear support vector machine to train a model of saliency. We compare the performance of saliency models created with different task-oriented attention and show that our approach performs better in predicting human visual attention regions than MIT model [3], which is one of the best models in predicting context-free human gaze.

The structure of this paper is as follows: Section 2 provides a brief description and discussion of some previous works.  Section 3 is devoted to describing the characteristics of the database. In Section 3.1, we present the data collection method, the images, eye tracking data, and ground truth data.  Section 3.2 analyzes the properties of our database. The detailed description of our model is in Section 4 that evaluates our approach using the popular saliency model evaluation scores (AUC) with MIT saliency model. The discussion and conclusions are discussed in the last section.

## 2. Related Work

Attention and saliency play important roles in visual perception. In past few years, more than two dozen of such databases are now available in the public domain. Fixations in Faces (FIFA) [10] were collected from eight subjects performing a 2 s long free-viewing task on 180 color natural images. It demonstrates the fact that faces attract significant visual attention. Subjects were found to fixate on faces with over 80% probability within the first two fixations. The NUSSEF database [11] was compiled from a pool of 758 images and 75 subjects. Each image was presented for 5 seconds and free-viewed by at least 13 subjects. A big feature of this dataset compared with others is that the 758 images in the dataset contain a large number of semantically affective objects/scenes such as expressive faces, nudes, unpleasure concepts, and interactive actions. MIT database from Judd et al. [12] included 1003 images collected from Flickr and LabelMe. Eye movement data were recorded from 15 users who free-view these images for 3 s. In this database, fixations were found around faces, cars, and text. Many fixations are biased towards the center. The DOVES dataset [13] includes 101 natural grayscale images [14]. Eye movements from 29 human observers as they free-view the images were collected. However, all of these databases record “free-viewing” eye movements. In addition, MIT CVCL Search Model Database [15] was recorded to understand task-oriented eye movement patterns of users. Observers were asked to perform a person detection task, and their eye movements were found to be consistent, even when the target was absent from the scene. This database was recorded based on task-oriented attention, but its task is single. So it is necessary to create a content-rich database based on task-oriented attention.

Several visual attention models are directly or indirectly inspired by cognitive concepts which are from psychological or neurophysiological findings. The winner-take-all (WTA) biologically plausible architecture which is related to the Feature Integration Theory is proposed by Koch and Ullman [16]. Built on WTA, Itti et al. [17] first implemented the computational model using a center-surround mechanism and hierarchical structure to predict salient regions. In this model, an image is predecomposed into low-level attributes such as color, intensity, and orientation across several spatial scales. The WTA inference pulls out the position with most conspicuity set of features. Later, Le Meur et al. [18] proposed a bottom-up coherent computational approach based on the structure of the human visual system (HVS), which used contrast sensitivity, perceptual decomposition, visual masking, and center-surround interaction techniques. It extracted features in Krauskopf's color space and implemented saliency in three separate parallel channels: visibility, perceptual grouping, and perception. A feature map is obtained for each channel, and then a unique saliency map is built from the combination of those channels. Based on the isotropic symmetry and radial symmetry operators of Reisfeld et al. [19] and the color symmetry of Heidemann [20], Kootstra et al. [21] developed three symmetry-saliency operators and compared them with human eye tracking data. E. Erdem and A. Erdem [22], Marat et al. [23], and Murray et al. [24] are other models guided by cognitive findings.

Another class of models is derived mathematically. Itti and Baldi [25] defined surprising stimuli as those which significantly change beliefs of an observer. This is modeled in a Bayesian framework by computing the KL divergence between posterior and prior beliefs. Similarly, Zhang et al. [26] proposed SUN (Saliency Using Natural statistics) model in which bottom-up saliency emerges naturally as the self-information of visual features. Bruce and Tsotsos [27] present a model for visual saliency built on a first principles information theoretic formulation dubbed Attention based on Information Maximization (AIM). Avraham and Lindenbaum's work on Esaliency [28] uses a stochastic model to estimate the most probable targets mathematically. Schölkopf et al. [29] proposed the Graph-Based Visual Saliency (GBVS) model, which used a Markovian approach to describe dissimilarity and concentration mass regions. Seo and Milanfar [30] and Liu et al. [31] are two other methods based on mathematical models.

Another class of models computes saliency in the frequency domain. Hou and Zhang [32] proposed Spectral Residual Model (SRM) by relating spectral residual features in spectral domain to the spatial domain. In [28], Avraham and Lindenbaum proposed Esaliency, a stochastic model, to estimate the probability of interest in an image. They roughly segmented the image first and used a graphical model approximation in global considerations to determine which parts are more salient.

Our proposed approaches are related to those models that learn mappings from recorded eye fixations or labeled salient regions. These models use some high-level features obtained from earlier databases and conduct learning mechanisms to determine model parameters. Torralba et al. [33] proposed an attentional guidance approach that combines bottom-up saliency, scene context, and top-down mechanisms to predict image regions likely to be fixated by humans in real-world scenes. Based on a Bayesian framework, the model computes global features by learning the context and structure of images, and the top-down tasks can be implemented in the scene priors. Cerf et al. [34] proposed a model that adds several high-level semantic features such as faces, text, and objects to predict human eye fixations. Judd et al. [12] proposed a learning-based method to predict saliency. They used 33 features including low-level features such as intensity, color, and orientation; midlevel features such as a horizon line detector; and high-level features such as a face detector and a person detector. The model used a support vector machine (SVM) to train a binary classifier. Zhao and Koch [35] proposed a model similar to that of Itti et al. [17], but with faces as an extra feature. Their model combines feature maps with learned weighting and solves the minimization problem using an active set method. Among the models described above, some focus on adding high-level features to improve predictive performance, while others use machine learning techniques to clarify the relationship between features and their saliency. However, the so-called high-level features are blur concepts and do not encompass all types of environments.

These saliency models have been used to characterize RoIs in free-viewing task, but their use in particular task has remained very limited. Recent results suggest that, during task-oriented visual attention, in which subjects are asked to find a particular target in a display, top-down processes play a dominant role in the guidance of eye movements [36–40]. However, the so-called top-down features are blur concepts and do not encompass all types of environments. Here, we exploit more informative concepts including low-level, target location, and center bias, using machine learning for eye fixation prediction.

## 3. Database of Eye Tracking Data

We collected a large database of eye tracking data to allow large-scale quantitative analysis of fixation points and gaze paths and to provide ground truth data for saliency model research [41]. Compared with several eye tracking datasets that are publicly available, the main motivation of our new dataset is for studying task-oriented visual attention, that is, where observers look while deciding whether a scene contains a target.

### 3.1. Data Gathering Protocol

#### 3.1.1. Participants

Fifteen participants, undergraduate and graduate volunteers aged 19–32 years (*μ* = 23.3, *σ* = 38.4) with uncorrected and corrected normal eyesight, voluntarily joined this experiment. All the participants were from the Northwestern Polytechnical University.

#### 3.1.2. Apparatus

Tobii TX300 eye tracker was used to record eye movements. We set the sampling frequency to 300 Hz. The eye tracker tolerates a certain extent of head movements, which allows the subjects to move freely and naturally in front of the stimulus. Freedom of head movement is at 65 cm, 37 × 17 (width × height), where at least one eye is within the eye tracker's field of view. Max head movement speed 50 cm/s stimuli were presented on a 23-inch wide screen TFT monitor. The screen size was 50.5 cm × 28.5 cm. Its screen response time was typically 5 ms and its resolution was set to 1920 × 1080.

#### 3.1.3. Materials

We randomly selected 1307 images from VOC2012 as the stimuli. The longest dimension (could be either width or height) of each image was 500 pixels and the other dimension ranged from 213 to 500 pixels. The images contained eight categories, namely, airplane, motorbike, bottle, car, chair, dog, horse, and person.

#### 3.1.4. Procedure

The 1307 images were separated into eight groups. Each group contained 100 images from the same categories and 70 images from the other categories (10 images were selected from each of these categories). All subjects sat at a distance of approximately 65 cm from the screen in a relatively quiet room. The images from each group were presented randomly with their original size in the middle of screen. Before the test, a five-point target display was used for calibration. To ensure high-quality tracking results, we checked the calibration accuracy after each of the groups. If the accuracy of the eye tracker was within about 1° visual angle, the subjects can continue the next group. Otherwise, the calibration will be carried out again. Subjects will be given different instructions for each of the groups. For example, for airplane group, subjects would be asked to find airplane in each picture, while a picture may have zero, one, or more airplanes. Subjects should find airplanes as more as possible in one image and switch to the next one through hitting the space key. To encourage the subjects to concentrate on looking for the target, we took two measures to improve authenticity of test. On the one hand, each group (above-mentioned eight groups) was equally divided into three small subsets. Subjects will spend less time to view the small subsets and pay more attention to the stimuli. On the other hand, after each subset, the subjects took a 2 min break and did a memory test: how many airplanes did you find?

### 3.2. Analysis of Dataset

#### 3.2.1. Consistency

**Figure 1:**
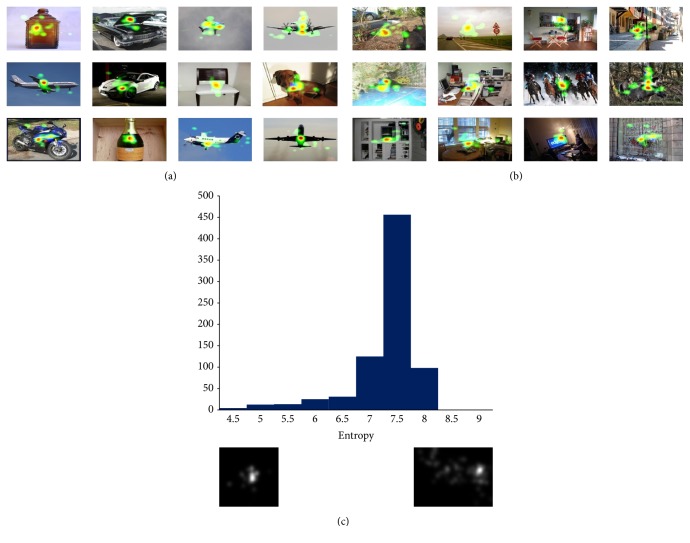
((a) and (b)) The heat map made from subjects gaze points with low and high entropy. If the image has high entropy, it usually contains more objects. (c) A histogram of the saliency map entropies.

In our dataset, for the target-present images, all subjects fixate on the same locations, while, in target-absent image, subjects' fixations are dispersed all over the image. We analyze this consistency of human fixations over an image by measuring the entropy of the average continuous saliency map across subjects. Though the original images were of varying aspect ratios, we resized them to 200 × 200 pixel images before calculating entropy.  Figure 1(c) shows a histogram of the entropies of the images in our database. It also shows a sample of 12 saliency maps (shown in Figures 1(a) and 1(b)) with lowest and highest entropy and their corresponding images.

#### 3.2.2. Center Bias

**Figure 2:**
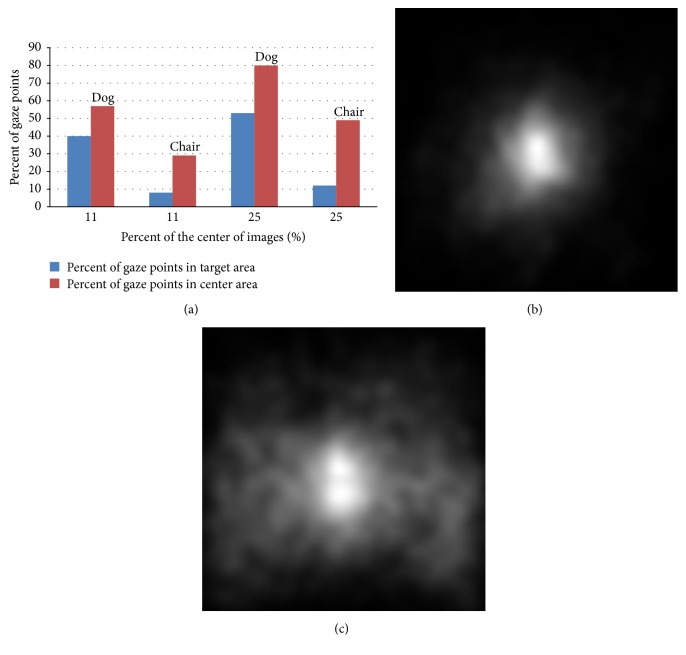
(a) The percentage of gaze points within the center 11% and 25% of the images, which is displayed by blue. Meanwhile, red shows the percentage of target gaze points. Obviously, in the dog category, the percentage of target gaze points is more than chair category. ((b) and (c)) Dog's and chair's average saliency map containing all the gaze points, which indicates a bias to the center of the image.

Our data indicates a strong bias for human fixations to be near the center of the image, as is consistent with previously analyzed eye tracking datasets [12, 42].  Figure 2 shows the average human saliency map separately from the dog and chair category, which have the strongest and weakest center bias. In the dog category, 57% of the gaze points lie within the center 11% of the image, and 80% of the gaze points lie within the center 25% of the image. In the chair category, 29% of the gaze points lie within the center 11% of the image, and 49% of the gaze points lie within the center 25% of the image.

There are several hypotheses for the root cause of center bias. In our test, the main reason is that people tend to place object or interesting things near the center of an image when taking a picture (the so-called photographer bias). To test this notion, we separately analyze percent of target gaze points, which are gaze points located on the target object within the center 11% and 25% of the dog and chair category. Obviously, in the dog category percentage of target gaze points in center area is more than that in the chair category. This difference has been attributed to the fact that target object mainly located on the center of images in dog category but was distributed in the whole image in chair category.

#### 3.2.3. Agreement among Observers

In this paragraph, we evaluate agreement of the fixation positions among observers. Analysis of the eye movement patterns across observers showed that the fixations were strongly constrained by the search task and the scene context. To evaluate quantitatively the agreement among observers, we studied the human interobserver (IO) model to predict eye fixations, under the same experimental conditions. The IO model outputs, for a given stimulus, a map built by integrating eye fixations from subjects other than the one under test while they watched that stimulus. Then the map was used to predict fixations of the excluded subject. Finally, we use the evaluation of IO model performance to evaluate the agreement among observers.

Using the area under the ROC curve (AUC) as the score, the IO model's map is treated as a binary classifier on every pixel in the image. Pixels with larger values than a threshold are classified as fixated while the rest of pixels are classified as nonfixated. Human fixations are used as ground truth. By varying the threshold, the ROC curve is drawn as the false positive rate versus true positive rate, and the area under this curve indicates how well the saliency map predicts actual human eye fixations.

We separately computed the IO model over 8 categories from our dataset and select the mean value as the result.  Table 1 shows the mean value of AUC scores of models. The results show that observers are very consistent with one another on the fixated locations in the target-present and target-absent conditions (over 85% in each case). On average, the agreement among observers is higher when the target is present than absent. This suggests that locations fixed by observers in target-present image are driven by the target location.

#### 3.2.4. Gaze Points in Each Stimulus

**Figure 3:**
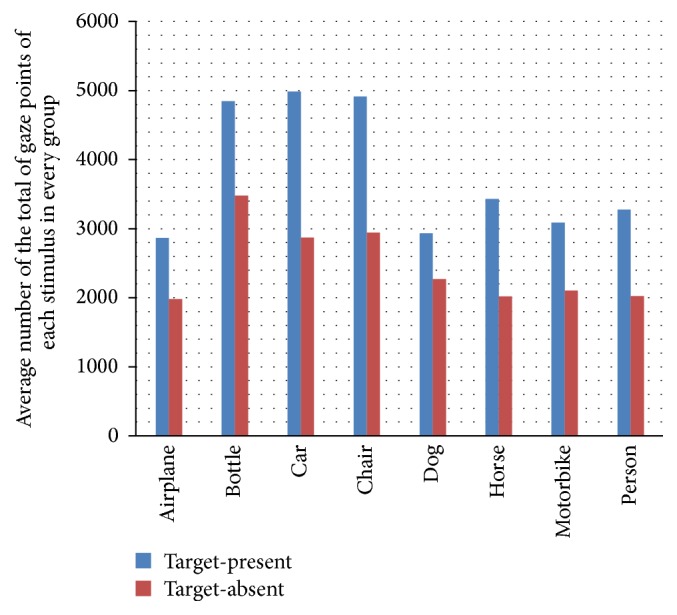
Average number of the total of gaze points of each stimulus in every group.

The task of counting target objects within picture is similar to an exhaustive visual search task. In our design, each scene could contain up to 4 targets. Target size was not prespecified and varied among the stimuli set. Under these circumstances, we expected observers to exhaustively search each scene, regardless of the true number of targets present.  Figure 3 shows the average number of the total of gaze points of each stimulus in every group. Unexpectedly, the count of fixations in the target-present is obviously more than target-absent.

**Figure 4:**
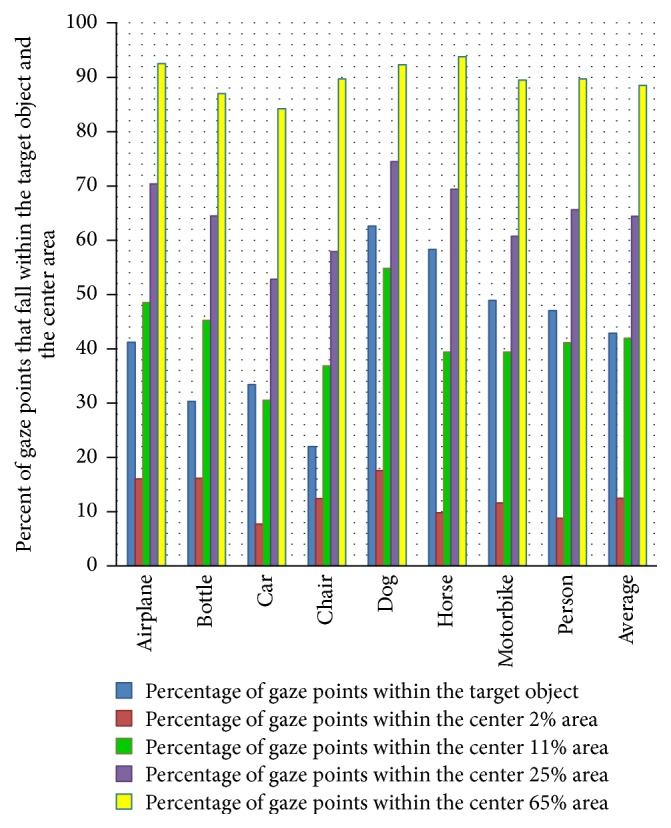
Percent of gaze points that fall within the target object and the center area.

To analyze the fixation position in the target-present images, we compare the percentage of human fixation that falls within the target object and the center area. In the first case, we apply the ground truth segmentation as the target object's area. In the second case, we calculate the percentage of human fixations located within the center 2%, 11%, 25%, and 65% of the image.  Figure 4 summarizes the results. First of all in two cases, the percentages both are above chance level. The differences seen in Figure 4 are statistically significant: the center 25% of the image better attracts human fixations than the target object area. This effect was mostly driven by subject's sidelong glance, for which human fixations are always around target object. But even so, the graphs in Figure 4 clearly indicate that the location of target object (the center area) and the area of target object will attract human fixations.

#### 3.2.5. Objects of Interest

According to Judd et al. [12, 42], if stimuli have one or more humans, gaze points should mainly locate on the human faces. However, in our test, this situation is not similar.

**Figure 5:**
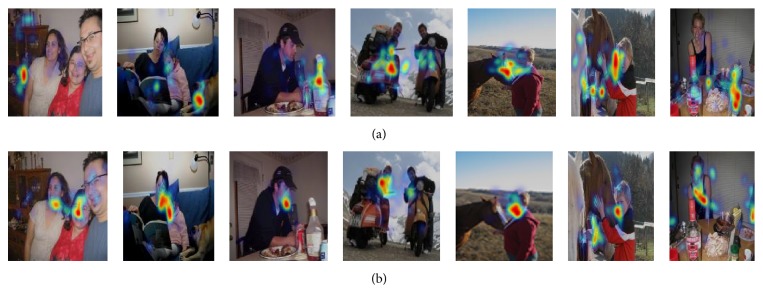
The figure shows the heat map of stimuli. (a) It shows the target-present's heat map but human is not target object. (b) It shows the target-present's heat map but human is target object.

Figure 5 shows heat map of stimuli in which have one or more humans. From Figure 5, we can know the following:

- For one stimulus, it has different heat map in different situation.
- If the human is the target object, many gaze points still locate on the human face.
- When subjects search target in the stimuli, they can ignore the other objects and pay all attention to the target object.

From what we have discussed above, we know that in our test whether some object is of interest depends on the task.

## 4. Learning-Based Saliency Model

**Figure 6:**
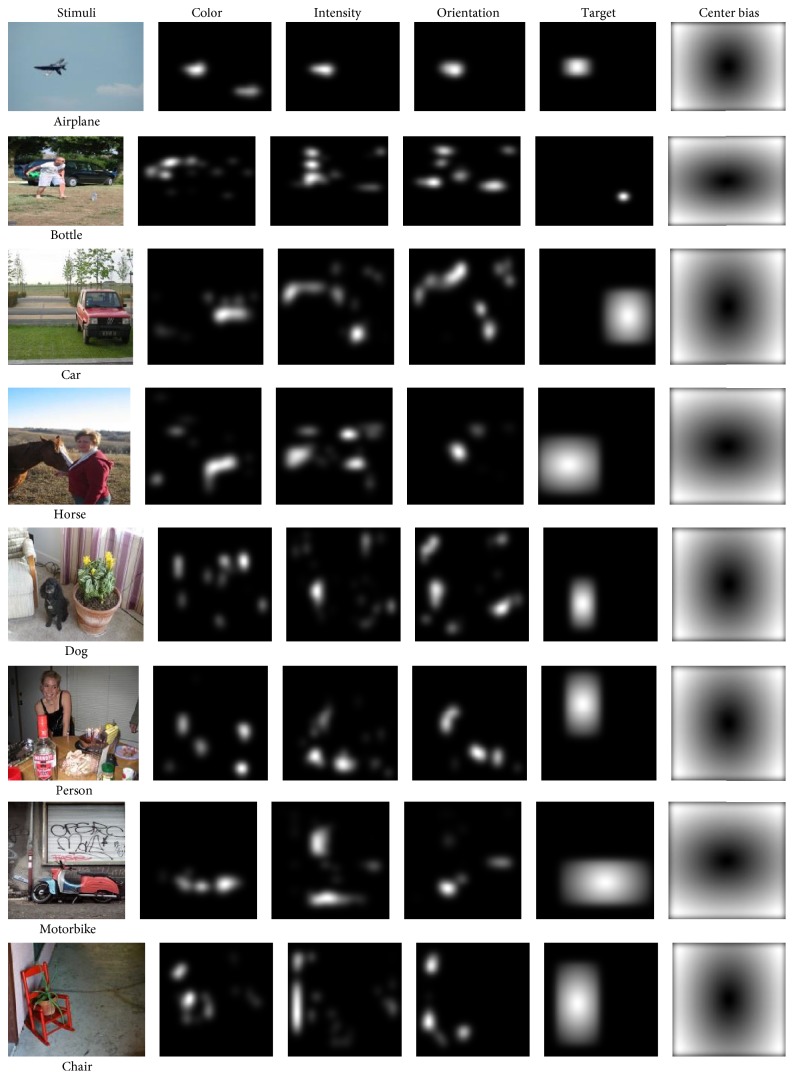
The figure shows the low-level feature maps such as color, intensity, orientation, and high-level feature maps such as the location of target object, finally, center-bias feature map.

In contrast to previous computational models that combine a lot of biologically plausible filters together to estimate visual saliency, we use a learning approach to train a classifier directly from human eye tracking data. For each image, we precomputed the feature maps for every pixel of the image resized to 200 × 200 and used the maps to train our model.  Figure 6 shows the feature maps. Through analyzing our dataset, we promoted low-level, high-level, and center prior features.

Low-level features, intensity, orientation, and color contrast have long been seen as significant features for bottom-up saliency. We include the three channels corresponding to these image features as calculated by Itti and Koch's saliency method [43]. Regarding high-level features, according to our data analysis, we found that humans gaze points always located on target object. So we used the location of target object as the high-level features. Firstly, bounding boxes around objects were labeled and we used them as the target object's area. Secondly, in the boxes, we used the distance of every pixel to the center of box instead of the pixel. Finally, out of boxes, we used zero instead of the pixel. Center bias, when humans take pictures, they naturally frame an object of interest near the center of the image. For this reason, we include a feature which indicates the distance to center of each pixel [12].

To evaluate our model, we followed the 5-fold cross validation method. The method partitions the database into five subsets randomly, each with *M* images. Every subset is selected sequentially as a test set and the remainders serve as the training set. Each time we trained the model from 4 parts and tested it over the remaining part. Results are then averaged over all partitions. From the ground truth gaze point map of each image, 20 pixels were randomly sampled from the top 20% salient locations, and 20 pixels were sampled from the bottom 70% salient locations to yield a training set of 3200 positive samples and 3200 negative samples. The purpose of choosing a 1 : 1 sampling ratio is to balance the distributions of positive and negative sample pixels in the same image. We chose samples from top 20% and bottom 70% in order to have samples that were strongly positive and strongly negative. The training samples were normalized to have zero mean and unit variance. The same parameters were used to normalize the test set.

We used the linear support vector machine [44] to train the model which was first used to learn the weight of each low-level, high-level, and center prior attribute in determining the significance in attention allocation. We used models with linear kernels because they are faster to compute, and the resulting weights of attributes are intuitive to understand. For each group, the average (Avg) and the corresponding standard deviations (STD) across the number of experiment executions of the learned weight of each attribute are shown in Table 2. It is clear that the attribute of center bias and the location of target object have the higher weight than others. Obviously, in the dog group, the weight of center bias is stronger than others. However, in the chair group, the weight of the location target object is stronger than others. For this phenomenon, the flowing may be critical. The areas of target object may contribute to the phenomenon. But we do not know the detailed relations. The weight of attribute also agrees with previous finding in figure-ground perception that, during visual search tasks, in which subjects are asked to find a particular target in a display, top-down processes play a dominant role in the guidance of eye movements.

## 5. Evaluation

**Figure 7:**
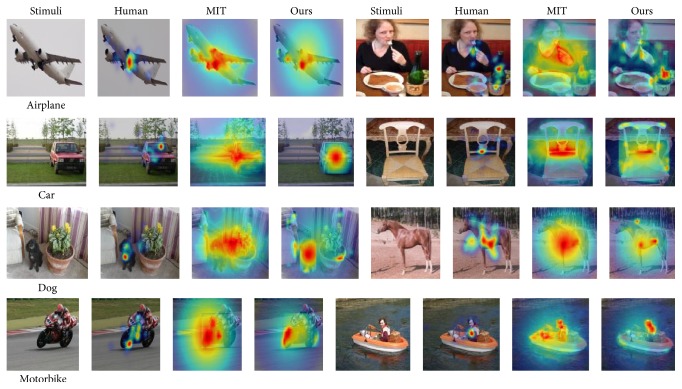
The figure shows the heat maps, which are generated by our model and MIT model. They were trained by the same gaze points and used the same training method.

To measure performance of saliency models, we performed comparisons of our models with the MIT model [3] which is one of the best models in predicting context-free human gaze. The model incorporated bottom-up saliency and high-level image semantics and works well in predicting saliency in a free-viewing context. To make the result comparable, the MIT model is trained on the same training set as our method.  Figure 7 shows heat maps of our model and the compared model. This is result for one image in each group. We conducted our experiment on 160 images randomly selected.

**Figure 8:**
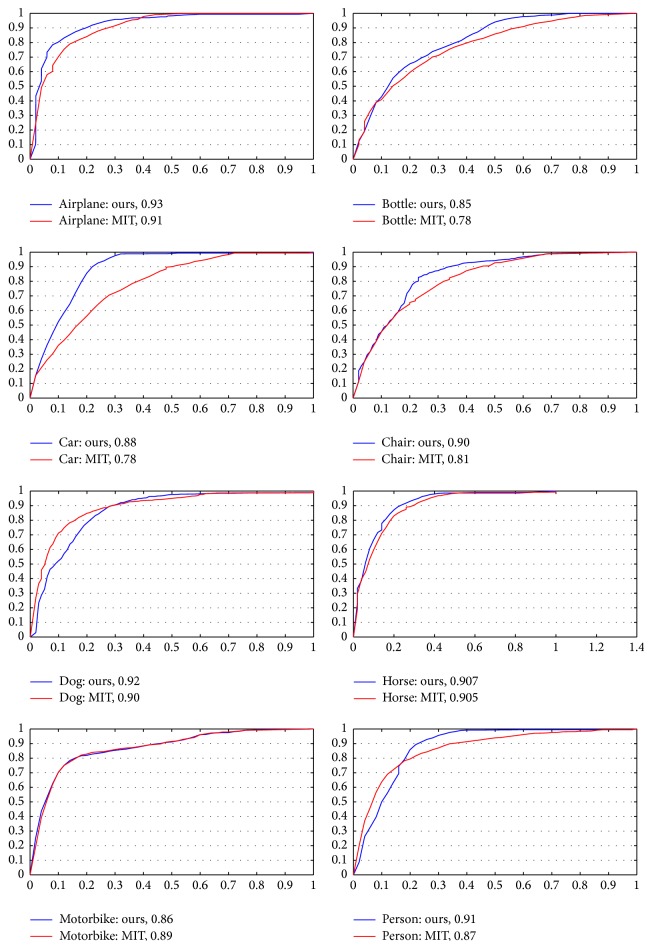
The figure shows the Receiver Operating Characteristic (ROC) for our model and MIT model. For each picture, the false alarm rate, on the *x*-axis, and the detection rate, on the *y*-axis. Besides, for each category, we calculate the averaging AUC scores of all the predictions, which are shown in above picture.

Figure 8 shows Receiver Operating Characteristic (ROC) for our model and MIT model. These curves show the proportion of gaze points that fall within the saliency map predicated by saliency model (detection rate) in relation to the proportion of the image area selected by the saliency map (false alarm rate). Our saliency models were generated by a weighted linear combination of the feature maps using the learned weights of each attribute. It shows how well the gaze points of each subject can be predicted by saliency model. For each category, we calculate the average (Avg) and the corresponding standard deviations (STD) across the number of experiment executions of the area under the ROC curve (AUC), which is shown in Table 3.

It can be seen that, for the MIT model, the performance is not always well; however, our model is better than MIT. For example, in bottle, car, and chair category, MIT model predicted gaze points regions with lower accuracy (AUC = 0.7881, AUC = 0.7865, and AUC = 0.8152) than our models (AUC = 0.8566, AUC = 0.8873, and AUC = 0.9015). From Table 3, we know that the weight of location of target object is first in the car and chair category. So, the promotion of accuracy mainly results from target guidance factor. However, even our model could not compete with human agreement.

## 6. Discussions and Conclusions

According to Figure 8 and Table 3, it is obviously shown that, for the bottle, car, and chair category, MIT model has lower performance, while our model has larger better performance than it. The main factor is that in these categories target object is small or not salient, so when subjects are free-viewing, they are not saliency map. However, in the task-oriented attention, they become the saliency map; that is why free-viewing model is not appropriately task-oriented.

As we all know several recent datasets [10–12, 45] all set the free-viewing time to 2–5 s per image. In our paradigm, the time was given to the subjects, which is mostly motivated by the following factors. If the viewing duration is too short, subjects might not have enough time to find the target objects and also promote the weight of center bias. On the other hand, if the viewing duration is too long, as the viewing proceeded, top-down or other factors (e.g., subjects feel bored and tired) come into play and gaze points become noisier. In addition, if the viewing duration is too long, gaze points may become the free-viewing.

Daily human activities involve a preponderance of visually guided actions, requiring observers to determine the presence and location of particular objects. Based on it, we researched how consistent human gaze points are across an image. Previous research and experience have shown that the gaze point location of several humans is strongly indicative of where a new subject will look, whether target-absent, and target-present. We implemented computational model for target-present in visual search and evaluated how well the model predicted subject's gaze points locations. In our experience, when subjects looked at a scene with a particular task, they consistently payed greater attention to the location of target objects and ignored the other saliency objects, such as text and people. So, our model combined the location of target as the high-level features. Ultimately, the model of attentional guidance predicted 95% of human agreement with the location of target object component providing the most explanatory power.

In this work we make the following contributions. We develop a collection of eye tracking data from the 11 people across 1307 images and have made it public for research use. It is the largest eye tracking database based on the visual search, which provides not only the accurate subjects' gaze points but also segmentation of target object for each image. In this search task, the location of target object is a dominating factor. We use machine learning to train a bottom-up, top-down model of saliency based on low-level, high-level, and center prior features. Finally, to demonstrate performance of our model, the same method was used to train MIT model.

For future work we may be interested in researching that the subjects' gaze points are tightly clustered in very small and specific regions, but our model selects a much more general region containing many objects without gaze points. We believe that the features of target object such as size, scale, and shape will lead subjects to fixate on target, which should be researched more carefully.

## Figures and Tables

**Table 1:** Intersubject agreement for target-present and target-absent.

Group name	Target-present	Target-absent
Airplane	0.90	0.90
Bottle	0.87	0.87
Car	0.86	0.86
Chair	0.83	0.84
Dog	0.95	0.95
Horse	0.94	0.94
Motorbike	0.93	0.93
Person	0.92	0.93
Average	0.90	0.92

**Table 2:** The table shows the average (Avg) and the corresponding standard deviations (STD) of the weight of attribute in each category. For every category, the bold weight is the first and the second is italic weight.

Category	Color		Intensity		Orientation		Target		Center bias	
	Avg	STD	Avg	STD	Avg	STD	Avg	STD	Avg	STD
Airplane	0.0319	0.00005	−0.0154	0.00002	0.0098	0.00002	*0.1201*	0.00012	**−0.4344**	0.00025
Bottle	0.0346	0.00006	0.0424	0.00006	0.0294	0.00004	*0.1206*	0.00009	**−0.3586**	0.00019
Car	0.0073	0.00001	0.0112	0.00002	−0.0159	0.00002	**0.2575**	0.00016	*−0.2418*	0.00012
Chair	0.0234	0.00003	0.0578	0.00006	0.1002	0.00011	**0.2766**	0.00013	*−0.1348*	0.00008
Dog	0.0066	0.00001	0.0075	0.00001	0.0848	0.00006	*0.1065*	0.00008	**−0.4556**	0.00024
Horse	0.0241	0.00004	−0.0004	0.00000	0.0240	0.00003	*0.1445*	0.00011	**−0.3182**	0.00031
Motorbike	−0.0088	0.00002	0.0166	0.00002	0.0276	0.00004	*0.2001*	0.00015	**−0.2733**	0.00025
Person	−0.0131	0.00003	−0.0291	0.00003	0.0638	0.00006	*0.1241*	0.00007	**−0.3159**	0.00027

**Table 3:** The table shows the average (Avg) and the corresponding standard deviations (STD) of the AUC in each category.

Model	Category							
	Airplane	Bottle	Car	Chair	Dog	Horse	Motorbike	Person
MIT								
Avg	0.8572	0.7881	0.7865	0.8152	0.8639	0.8583	0.8563	0.7962
STD	0.0016	0.0012	0.001	0.0015	0.0006	0.0008	0.0013	0.0011
Ours								
Avg	0.8635	0.8566	0.8873	0.9015	0.8665	0.8663	0.8563	0.8893
STD	0.0012	0.0006	0.0005	0.0004	0.0006	0.0009	0.0007	0.0007
